# Nitrogen Reduction Testing with Real-Time ^15^NH_3_ Yield Quantification Using Orbital Multiturn Time-of-Flight Mass Spectrometry

**DOI:** 10.1021/acsenergylett.4c02961

**Published:** 2024-11-08

**Authors:** Logan M. Wilder, Kabirat Balogun, W. Ellis Klein, Prithviraj Chumble, James L. Young

**Affiliations:** † National Renewable Energy Laboratory, Golden, Colorado 80401, United States

## Abstract

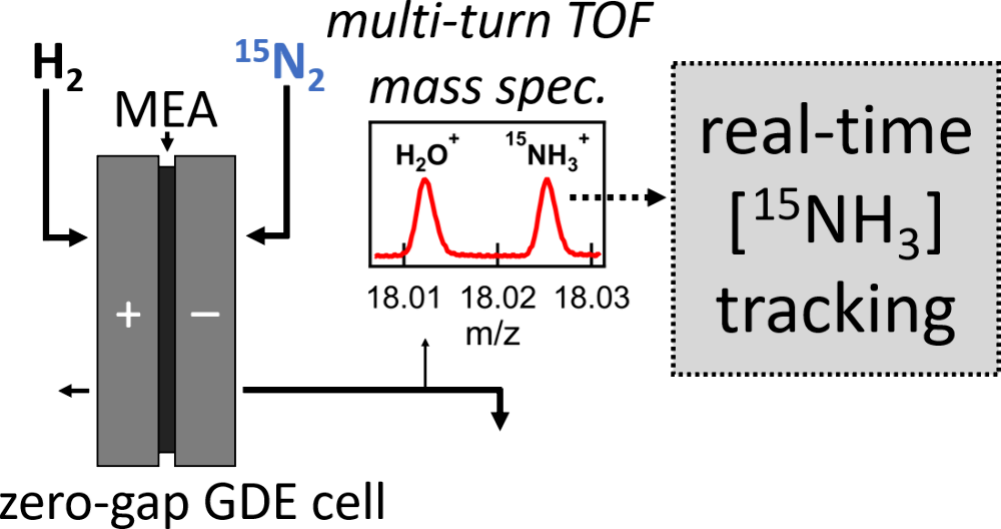

Yield validation of dinitrogen reduction reaction (N_2_RR) catalysts with ^15^N isotope labeling experiments is frustrated by the high cost of ^15^N_2_ and the frequent occurrence of ^15^NH_3_ and ^15^NO_*x*_ impurities in commercial ^15^N_2_ sources. Also, gas diffusion electrode (GDE) cell architectures are relevant to scaling N_2_RR but underexplored and limited by ex situ product analysis methods. To overcome these obstacles, we develop and demonstrate a protocol for N_2_RR catalyst testing using a scalable GDE cell architecture and specialized test station that facilitates in-line product analysis with multiturn time-of-flight mass spectrometry. This approach provides robust yield measurements through real-time monitoring of ^15^NH_3_ with ultrahigh mass resolution. The ^15^N-containing impurities are also monitored, allowing their influence to be mitigated. A minimum detectable yield of 6 pmol·cm^–2^·s^–1^ (at 2.00 sccm and utilizing a 5 cm^2^ electrode) for real-time ^15^NH_3_ yield rate measurements is achieved while using a cost-effective amount of ^15^N_2_ (<100 mL).

Advancement of the electrochemical dinitrogen reduction reaction (N_2_RR) for NH_3_ generation promises sustainable NH_3_ production, but fundamental and technical challenges hinder development.^1−3^ The fundamental challenges of N_2_RR for NH_3_ production include breaking the strong N≡N bond; low solubility of N_2_ in most solvents; and competition from side reactions, primarily the hydrogen evolution reaction (HER).^4^ Due to low NH_3_ yields at benchtop scale, the study of N_2_RR is complicated by the relatively high background of NH_3_ and labile N-containing compounds which are easily reduced to NH_3_ (for example, NO_*x*_). The most important experiment for validating N_2_RR NH_3_ yields is an isotope-labeling control, wherein yields are compared when ^15^N_2_ is reacted instead of ^14^N_2_. Isotope-labeling control experiments using ^15^N_2_ face unique challenges due to the high cost of ^15^N_2_ and frequent occurrence of ^15^NH_3_ and ^15^NO_*x*_ impurities in commercially available ^15^N_2_ sources.^5^ A possible solution to several of these fundamental and experimental-design challenges is the combination of a gas-diffusion electrode (GDE) architecture for N_2_RR electrolyzers with continuous monitoring of reactants (^14^N_2_, ^15^N_2_, ^14^NO_*x*_, ^15^NO_*x*_) and products (^14^NH_3_, ^15^NH_3_) using in-line mass spectrometry.

In GDE cell architectures, gas-permeable electrodes are situated on either side of a polymer or a liquid electrolyte. A schematic illustration of the zero-gap (polymer electrolyte membrane) GDE cell architecture used here is shown in Scheme 1. In comparison to the traditional liquid-submerged electrode H-cell architecture, a zero-gap GDE cell architecture presents four main advantages for studying the N_2_RR:

(1) The reaction occurs at a gas/solid interface, circumventing the low liquid solubility of N_2_ which limits local pN_2_ and can lead to mass transport losses at high current density.^6^
(2) Solid electrolytes (e.g., polymer membranes) allow for operation at variable humidity via the humidified supply gas, which can enable control of water activity, and indirectly, proton activity.^7^ The membrane used here is the cation exchange membrane Nafion 211.
(3) Produced NH_3_ is formed at the gas/electrode phase boundary and can readily leave the cell in the gas phase, facilitating its real-time detection in the absence of liquids that have high NH_3_ solubility.
(4) The use of a zero-gap GDE architecture eliminates the need for purging expensive ^15^N_2_ into a liquid electrolyte. The ^15^N_2_ control experiments demonstrated here use only 1.00 sccm ^15^N_2_ for <1 h, drastically lowering the cost of this critical control experiment in comparison to experiments that require continuous gas purging in liquid electrolyte.^8^

**Scheme 1:**
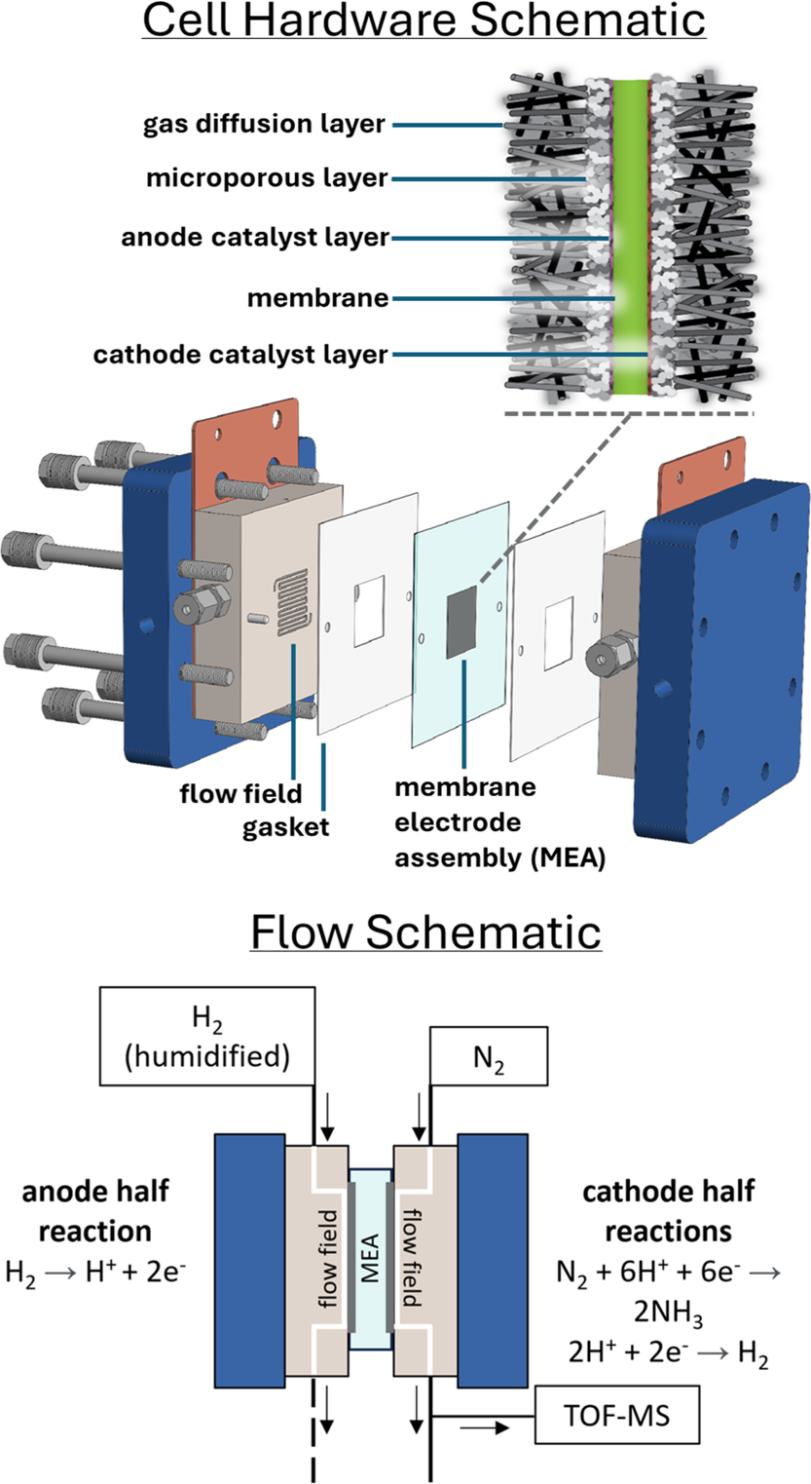
Schematics of the N_2_RR Electrochemical Cell Hardware Featuring a Zero-Gap, Liquid-Free Design to Facilitate Real-Time Effluent Gas Analysis The membrane electrode assembly (MEA) in the top inset consists of a pair of gas diffusion electrodes sandwiching a polymer membrane. The bottom flow schematic illustration shows the path of gas flow through the cell and location of TOF-MS sampling.

Owing to the clear benefits of testing catalysts in a GDE cell architecture, which include high local pN_2_, humidity control, relevance to industry-scale N_2_RR, and comparatively lower cost of the critical ^15^N_2_ control experiment, there is a clear need for development of robust catalyst test methods in GDE cell architectures. Here, an in-line gas analysis method using multiturn time-of-flight mass spectrometry (TOF-MS) is developed and demonstrated. In general, quantification by MS has ultralow detection limits in comparison to typical UV–vis or NMR assays, as well as precise and sensitive detection of multiple products on the time scale of seconds.^9−11^ Multiturn TOF-MS, in particular, is uniquely suited for quantification in N_2_RR catalyst testing experiments, as it enables high mass resolution and can resolve chemical species with the same nominal mass. Specifically, the ability to resolve ^15^NH_3_ and H_2_O, with exact masses differing by only 0.013 amu, is paramount.

## Testbed Design for Analysis of NH_3_ in the Gas Phase

The electrochemical N_2_RR test station and cell hardware demonstrated herein are designed to facilitate real-time, gas phase quantification of trace levels of NH_3_ (tens to hundreds of pmol·s^–1^ at 2.00 sccm). Design criteria include minimization of the internal surface area to which NH_3_ is exposed, use of surface-treated gas lines to minimize NH_3_ adsorption,^12^ and use of a gas purifier to remove nitrogenous impurities from the gas supply.^5^ The relevant gas handling tubing is coated with hydrogenated amorphous silicon (Sulfinert, Restek), and electropolished stainless steel is used for all fittings. A diagram of the test station design showing the specific component materials used is provided in the Supporting Information (Figure S1).

To remove NH_3_ and NO_*x*_ from the ^14^N_2_ and ^15^N_2_ feed gases, the test station includes a gas purifier with powder absorbent media (UltraPure Mini PF, NuPure). Analysis of the ^15^N_2_ gas source used in this study (Supplier: Cambridge Isotope Laboratories) revealed a ^15^NH_3_ concentration of 60 ± 20 ppm (note: the “ppm” unit reported here is a gas phase concentration, which reflects a total NH_3_ quantity per volume about 1,200× lower than a liquid phase ppm concentration).^13^ The ^15^NH_3_ concentration in the ^15^N_2_ source gas is significantly reduced when the purifier is engaged, as shown in Figure S2 of the Supporting Information.

## Tuning Multiturn TOF-MS Parameters for NH_3_ Detection

**Figure 1:**
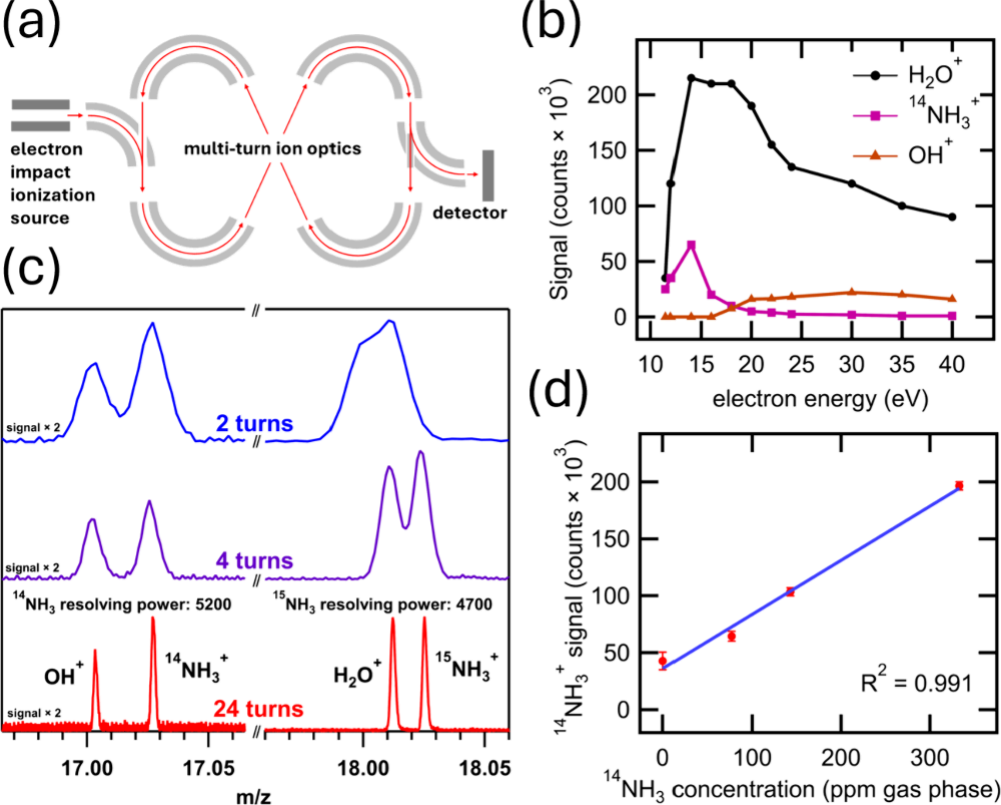
(a) Schematic of the orbital multiturn time-of-flight mass analyzer ion optics showing the path ions travel during one full turn (red line), (b) signal intensity of H_2_O^+^, ^14^NH_3_^+^, and OH^+^ ions as a function of electron impact ionization (EI) electron energy, (c) mass spectra of H_2_O and NH_3_ in N_2_ carrier gas demonstrating baseline resolution between the OH^+^ and ^14^NH_3_^+^ peaks and between the H_2_O^+^ and ^15^NH_3_^+^ peaks at 24 turns (red trace), and (d) calibration curve showing ^14^NH_3_^+^ signal as a function of ^14^NH_3_ concentration. For illustrative purposes in panel c, a higher electron energy of 20 eV is used for the spectra on the left (*m*/*z* = 16.97–17.06) than the 11.5 eV energy used on the right (*m*/*z* = 17.96–18.06) and elsewhere in this work. The error bars in panel d represent the standard deviation of three replicate measurements of the calibration curve on three separate days in a three-day period.

Key to applying mass spectrometry for isotope-selective N_2_RR analytics is the ability to fully resolve H_2_O^+^ (18.011 amu) and ^15^NH_3_^+^ (18.024 amu). Notably, N_2_RR product analysis by mass spectrometry was demonstrated by Krempl and co-workers^14^ using a mass analyzer with unit mass resolution. However, this relatively low mass resolution limited the approach to observation of the ^14^NH_3_^+^ signal (17.027 amu), which in some cases can be convoluted with the OH^+^ fragment (17.003 amu) of H_2_O. In contrast, ultrahigh mass resolution can be achieved with multiturn TOF mass analyzers that enable long ion flight path lengths (e.g., 1–100 m) in a compact space. Furthermore, the orbital multiturn analyzer used here provides a selectable flight path length. An illustration of the orbital multiturn ion optics in the JEOL InfiTOF (JEOL, model # JMS-MS3010HRGA) mass spectrometer used in this study is shown in Figure 1a. The path length is established by the number of orbital loops, referred to as “turns”, that ions travel before reaching the detector. Ions are injected into the analyzer (Figure 1a left side) and, after a specified number of turns, are ejected to the detector (Figure 1a right side).

The gas stream of interest is continuously sampled via a capillary and ionized by electron impact ionization (EI). Figure 1b shows the signal intensity of the H_2_O^+^, ^14^NH_3_^+^, and OH^+^ peaks as a function of EI electron energy in the range of 11.5–40 eV. The ^14^NH_3_^+^ signal shows a local maximum at 14 eV, which can be explained by the onset of NH_3_ ionization at 10.4 eV and by the signal decrease corresponding to the onset of NH_3_ fragmentation at 14.8 eV.^15^ We chose to use an electron energy of 11.5 eV to minimize analyte fragmentation and maximize the ratio of the ^15^NH_3_^+^ peak intensity versus the H_2_O^+^ peak intensity, which, in addition to the ultrahigh mass resolution achieved here, prevents overlap of the ^15^NH_3_^+^ and H_2_O^+^ peaks.

The mass spectra in Figure 1c illustrate the resolving power for 2-, 4-, and 24-turn flight path lengths. The 2-turn path is not sufficient to resolve H_2_O^+^ and ^15^NH_3_^+^; however, increasing to 4 turns partially resolves these species, and 24 turns (representing a total path length of ∼24 m) achieves clear baseline resolution between H_2_O^+^ and ^15^NH_3_^+^ as well as between OH^+^ and ^14^NH_3_^+^.

The signal intensity of the ^14^NH_3_^+^ and ^15^NH_3_^+^ ions at 0 ppm, that is, in conditions with no added ^14^NH_3_ or ^15^NH_3_, had some day-to-day variation, which is attributed to variation in background NH_3_ concentration and variation in the system base pressures. To account for this, NH_3_ yield rates during catalyst testing are normalized to the baseline signal and reported as ΔNH_3_. During experimental runs, after a period of stabilization of the instrument, the background NH_3_^+^ signal showed little variation (<2% average_30 min_ signal change over 1 h, Figure S3). Moreover, the background NH_3^+^_ signal can be measured before and after an N_2_RR test, allowing for the baseline signal drift to be observed and corrected if needed. A calibration curve for ^14^NH_3_ is shown in Figure 1d. The calibration curve-derived limit of detection (LOD) is determined to be 910 pmol·cm^–3^ (27 ppm, gas phase), which corresponds to an NH_3_ yield of 6 pmol·cm^–2^·s^–1^ (with a 2.00 sccm effluent flow rate and 5 cm^2^ electrode, which is typical in this study). In comparison to the commonly used nuclear magnetic resonance (NMR) method for isotope-selective ^15^NH_3_ quantification,^11,16^ this multiturn TOF-MS method represents a ∼10× improvement in LOD while adding the significant robustness of real-time tracking of ^15^NH_3_ and potential sources of false positive results such as labile ^15^N-containing impurities.

**Figure 2:**
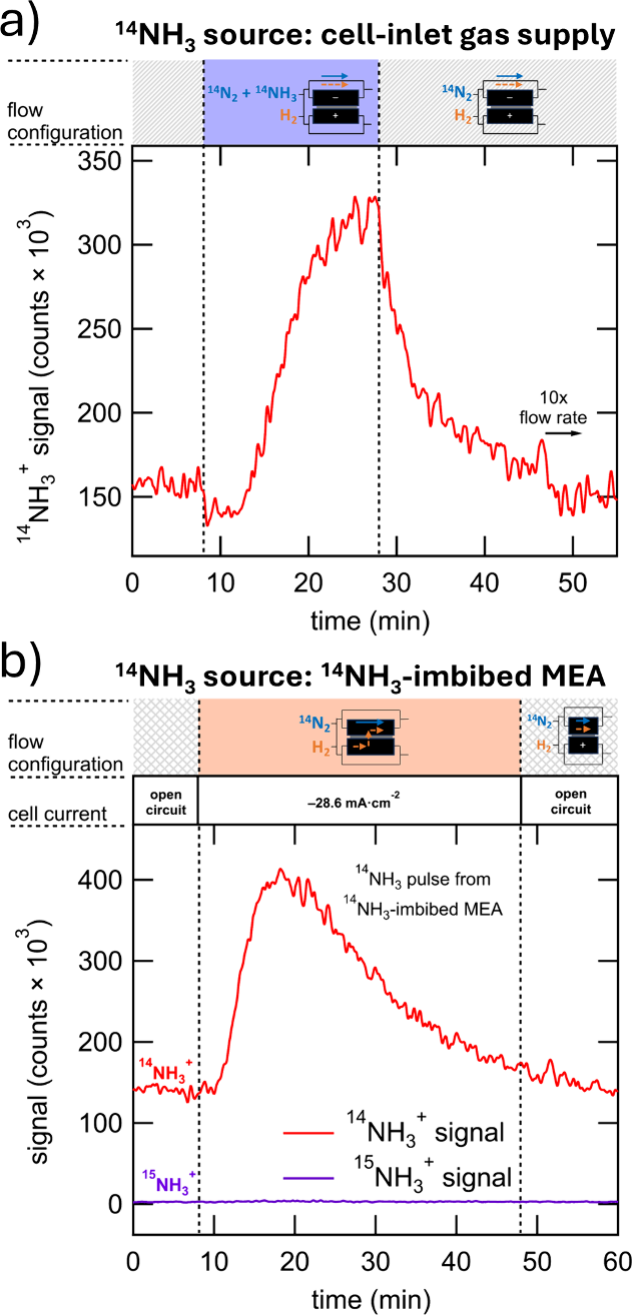
Single-ion chromatograms of ^14^NH_3_^+^ showing the response to a ^14^NH_3_ dose from either (a) the cell inlet gas supply with the cell hardware bypassed or (b) an MEA imbibed with NH_3_. In panel a, the initial condition is a 1:1 mixture of ^14^N_2_ and H_2_ gas flowing at 2.00 sccm into the test station. At *t* = 8–28 min, NH_3_ (500 ppm gas phase) is added to the gas mixture, simulating an NH_3_ yield rate of 560 pmol·s^–1^. The flow rate is increased to 20.0 sccm at *t* = 47 min to purge the test station. In panel b, the initial condition is a 1:1 mixture of ^14^N_2_ and H_2_ gas flowing at 2.00 sccm into the cathode-side flow field of the cell hardware. At *t* = 8 min, a constant current of −28.6 mA·cm^–2^ is applied, which pulls the NH_3_ from the MEA via electroosmotic drag, where it then readily travels through the GDE, into the flow field, and to the TOF-MS for detection.

Two control experiments are conducted to assess the responsiveness of the test station to NH_3_ (Figure 2). First, to assess the responsiveness of the test station to a defined ^14^NH_3_ pulse, trace ^14^NH_3_ in a 1:1 mixture of ^14^N_2_ and H_2_ (1.00 sccm each) is introduced into the test station while measuring the ^14^NH_3_^+^ ion signal. The 1:1 mixture of ^14^N_2_ and H_2_ is used to simulate an N_2_RR cell effluent gas mixture with a significant amount of H_2_ present as a result of the HER side reaction during catalyst testing. The results of this test are shown in Figure 2a. A clearly elevated response is observed <10 min after ^14^NH_3_ (500 ppm gas phase, simulated yield rate of 560 pmol·s^–1^) is introduced to the test station at *t* = 8 min. At *t* = 28 min, the gas flow is switched back to a 1:1 mixture of ^14^N_2_ and H_2_, and the ^14^NH_3_^+^ signal immediately decreases, reaching 1/e of the signal peak within <5 min. This short signal decay time is a favorable result in comparison to the previously discussed study by Krempl et al.,^14^ which relied on diffusion of NH_3_ from liquid electrolyte to gas phase for analysis and resulted in signal decay times >3 h. At *t* = 47 min, the flow rate is increased by a factor of 10, from 2.00 to 20.0 sccm, to purge lingering ^14^NH_3_ from the test station, and the ^14^NH_3_^+^ signal returns to its starting level in <5 min. Second, an additional test is conducted (Figure 2b) to assess the responsiveness of the full system to ^14^NH_3_ that originates within the membrane electrode assembly (MEA). In this test, the membrane was first imbibed with an aqueous solution of ^14^NH_3_ (25% v/v) before the MEA and the cell were assembled. During this simulated N_2_RR test, a constant current of −28.6 mA·cm^–2^ is applied, which pulls the NH_3_ from the membrane to the catalyst layer via electroosmotic drag where it then readily travels through the GDE, into the flow field, and to the TOF-MS for detection.^17^ It is thus clear from Figure 2b that NH_3_ present or generated within the MEA rapidly exits the cell and reaches the TOF-MS for detection. It follows that our analytical approach and test system is compatible with other methods of N_2_RR that produce gas-phase NH_3_, such as the Li-mediated N_2_RR approach demonstrated by Li et al.^18^ where 98% of the produced NH_3_ was readily transferred to the gas phase.

## In-Line ^15^N Isotope Tracing Protocol

To assess the effectiveness of the N_2_RR catalyst test bench and demonstrate a catalyst testing protocol, we monitor the ^15^NH_3_ yield of a sputter-deposited Ru (S–Ru) catalyst under a ^15^N_2_ supply. Several studies report moderate-to-exceptional N_2_RR activity and faradaic efficiency (FE) on Ru-based catalysts,^19−21^ including work by Wei et al.^22,23^ specifically in a GDE cell architecture. In contrast, a study by Kolen et al.^24^ did not report significant N_2_RR activity in a screening of several Ru-containing bimetallic alloy catalysts in a GDE cell architecture. The apparently promising activity of Ru-based catalysts motivated us to apply S–Ru as a model catalyst in this work. To prepare the S–Ru GDE, Ru is sputter-deposited onto a gas diffusion layer (GDL) material (Sigracet 29BC, SGL Carbon) consisting of a microporous carbon catalyst support layer and a carbon fiber gas diffusion layer (Scheme 1). The Ru loading is 0.12 mg·cm^–2^ as determined by X-ray fluorescence spectroscopy. The S–Ru catalyst is characterized by X-ray photoelectron spectroscopy (XPS) before and after catalyst testing (Figure S4). Characterization by XPS reveals the as-deposited S–Ru catalyst surface initially shows a mixture of Ru^0^ and RuO_*x*_.^25^ Following catalyst testing, the amount of RuO_*x*_ component is reduced. Details of the analysis of the S–Ru XPS characterization are included in the Supporting Information. The anode catalyst is sputter-deposited Pt on the same GDL type.

To test the S–Ru catalyst for N_2_RR activity, a protocol is defined and performed to determine if the S–Ru electrocatalytic system generates measurable NH_3_ during a current hold, here termed the isotope tracing protocol (ITP). To avoid the high natural background of ^14^NH_3_, ^15^N_2_ is used during the entirety of the ITP experiment. A current hold of −28.6 mA·cm^–2^ for a 5 cm^2^ active area is used in this demonstration, which conveniently corresponds to a theoretical H_2_ yield rate of 1.00 sccm assuming only trace amounts (<0.1% FE to NH_3_) of ^15^NH_3_ are produced. During the current hold, the cell voltage was stable between −0.334 V and −0.322 V, which is a voltage range similar to that reported by Wei et al.^22^ for a Ru-based catalyst in a GDE cell architecture that showed exceptional FE to NH_3_ (>60%). The ITP is carried out in zero-gap cell configuration, which uses a polymer membrane (Nafion 211) as the electrode separator and the solid-phase electrolyte that readily conducts protons when humidified. This zero-gap configuration is a significant difference in comparison to the study by Wei et al., which employed liquid electrolyte. Another difference is the form of Ru, which herein is sputter-deposited Ru, while Wei et al. used Ru nanoparticles supported on carbon black.

To avoid changes in the bulk composition of the cathode effluent gas, the ratio of H_2_ to ^15^N_2_ before and after the current hold is matched to the expected ratio of H_2_ to ^15^N_2_ during the current hold. The motivation for keeping a constant H_2_:N_2_ ratio throughout the test is to eliminate the influence of a varying H_2_:N_2_ ratio on the measured NH_3_^+^ signal. While this approach of using a fixed H_2_:N_2_ ratio is not strictly required, a variable H_2_:N_2_ ratio would require an NH_3_ calibration curve to be generated for each H_2_:N_2_ ratio. Such reliance on multiple calibration curves to calculate the NH_3_ yield could introduce the potential for error. Our method of using a constant H_2_:N_2_ ratio discerns a positive response from a null response without a calibration curve and requires only one calibration curve to compute the yield from a positive response. Further discussion of the advantages of maintaining a constant carrier gas N_2_:H_2_ ratio during catalyst testing is provided in the Supporting Information, and calibration curves showing the effect of varying H_2_ in the carrier gas with or without humidification are shown in Figure S5. Additionally, Figure S6 shows an example of a potentially misleading ^15^NH_3_^+^ signal response when the composition of the cell effluent is allowed to vary between pure ^15^N_2_ (cell at open circuit) and 1:1 ^15^N_2_:H_2_ (−28.6 mA·cm^–2^).

The ITP is demonstrated at a constant current density that gives a total cell current of −143 mA, which corresponds to an H_2_ production rate of 1.00 sccm with the initial assumption of trace ^15^NH_3_ production (FE to ^15^NH_3_ ≤ 1%). Accordingly, we feed 1.00 sccm ^15^N_2_ into the cell during operation such that the TOF-MS continuously samples a cell effluent of 1:1 ^15^N_2_:H_2_ that may contain trace ^15^NH_3_. To evaluate N_2_RR activity over a range of current densities, the 1:1 ^15^N_2_:H_2_ ratio can be maintained by matching the ^15^N_2_ supply flow rate to the H_2_ production rate. For example, the ^15^N_2_ supply flow rate should be increased to 2.00 sccm if the cell is operated at a total current of −286 mA (57.2 mA·cm^–2^), which corresponds to a 2.00 sccm H_2_ production rate. We initially assume that the ^15^NH_3_ generation FE is low (≤1%), which is later validated. In cases where the ^15^NH_3_ generation FE is significant (≥1%) and thus the H_2_ FE is ≤99%, the H_2_ concentration in the effluent should be tracked by measuring the total cell effluent flow rate in addition to the composition. The calibration protocol would then include collecting a set of calibration curves that span the range of N_2_:H_2_ ratios of the cell effluent. An example of the influence of varying the N_2_:H_2_ ratio on the calibration curve shape is shown in Figure S5.

**Figure 3:**
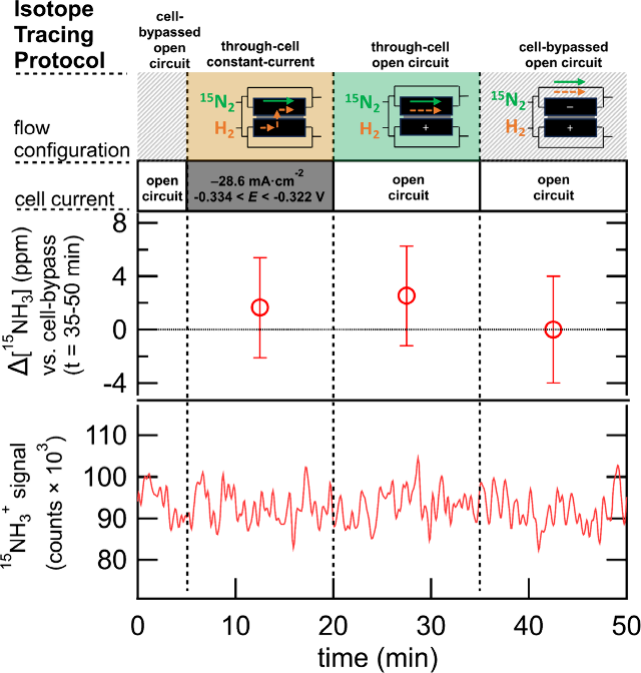
N_2_RR testing of sputter-deposited Ru (S–Ru) following the isotope tracing protocol (ITP). The bottom plot shows the single-ion chromatogram of ^15^NH_3_^+^ during the test. The upper plot shows Δ[^15^NH_3_] (concentration change) based on the average ^15^NH_3_^+^ signal during each test condition. The Δ[^15^NH_3_] during the *through-cell constant-current* (*t* = 5–20 min) and *through-cell open circuit* (*t* = 20–35 min) conditions is calculated relative to the control condition, *cell-bypassed open circuit* (*t* = 35–50 min).

Three test conditions are defined in the ITP: (1) gas flow bypassing cell at open circuit; (2) gas flow through cell at open circuit; and (3) gas flow through cell at constant current. The flow configuration of each test condition is described in Table 1 and is displayed in the top panel of Figure 3. The *cell-bypassed open circuit* condition represents the background ^15^NH_3_^+^ signal arising from ^15^NH_3_ in the ^15^N_2_ source gas, and in this condition, a 1:1 mixture of ^15^N_2_ and H_2_ flows through the cathode-side bypass line. The *through-cell open circuit* condition represents the background ^15^NH_3_^+^ signal arising from ^15^NH_3_ in the ^15^N_2_ source gas after interacting with interior components of the cell hardware, which may uptake or release NH_3_. In this condition, a 1:1 mixture of ^15^N_2_ and H_2_ flows through the cathode-side flow field. Finally, the *through-cell constant-current* condition represents the conditions under which the N_2_RR may occur. In this condition, ^15^N_2_ flows through the cathode-side flow field, H_2_ flows through the anode-side flow field, and a constant current of −28.6 mA·cm^–2^ is applied. The value of −28.6 mA·cm^–2^ also generates a cathode effluent flow of a 1:1 mixture of ^15^N_2_ and H_2_ if the FE of the tested catalyst toward HER is 100%. In this ITP test of the S–Ru catalyst, the cell voltage varies between −0.334 V and −0.322 V when operated at −28.6 mA·cm^–2^. The results of the ITP testing of S–Ru are shown in Figure 3.

**Table 1:**
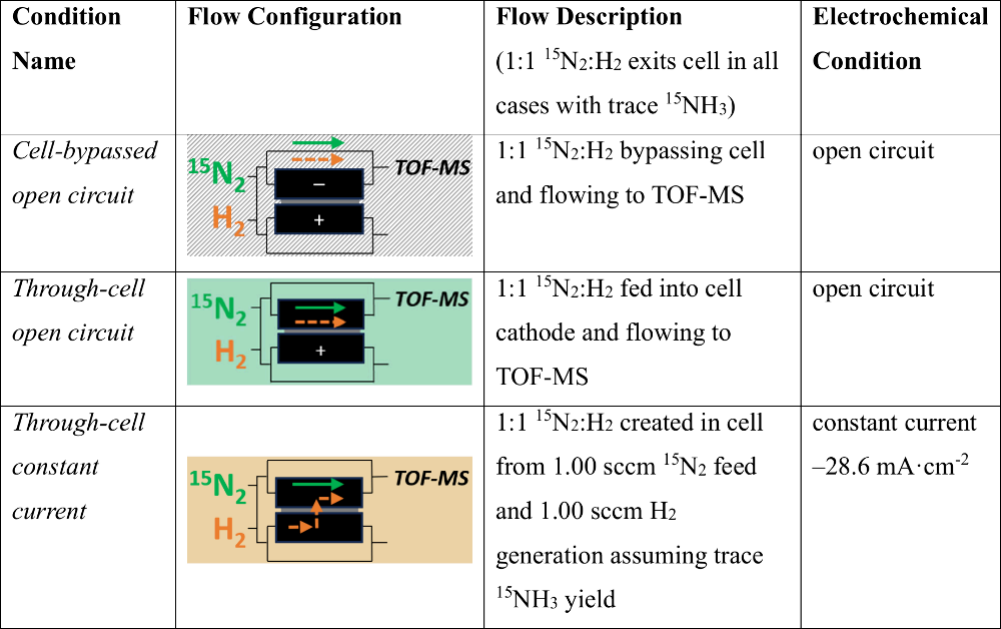
Isotope Tracing Protocol (ITP) Conditions

The bar graph in Figure 3 (top half) shows the difference in effluent ^15^NH_3_ concentration, denoted Δ^15^NH_3_, between the *through-cell constant-current* or *through-cell open circuit* conditions versus the *cell-bypassed open circuit* baseline condition. Comparison of the ^15^NH_3_ concentration during the *through-cell constant-current* condition to the baseline condition indicates that electrochemically generated ^15^NH_3_ is not detected in the S–Ru catalytic system. Further, comparison of ^15^NH_3_ concentration during the *through-cell open circuit* condition to that during the baseline condition indicates that the cell hardware does not uptake or release ^15^NH_3_ during the experiment. For context, if the FE to ^15^NH_3_ of the S–Ru catalyst was 1.0%, at −28.6 mA·cm^–2^ (with 2.00 sccm flow rate and 5 cm^2^ electrode surface area), the expected increase from the baseline would be 4.9 nmol·s^–1^ (4400 ppm, gas phase). Moreover, the minimum detectable yield based on the LOD of the method is 30 pmol·s^–1^ (27 ppm, gas phase, at 2.00 sccm flow rate) which corresponds to an FE of only 0.006%. It is clear from the data in Figure 3 that the selectivity to NH_3_ of the S–Ru catalyst tested here in the stated conditions is <0.006%. To show the reproducibility of the measurement, three replicates of the ITP are performed (Figure S7), which are consistent with the data in Figure 3.

In summary, a protocol is demonstrated for validating N_2_RR catalysts using in-line detection of ^15^NH_3_ in the effluent of a zero-gap, GDE cell using multiturn TOF-MS. A minimum detectable yield of 6 pmol·cm^–2^·s^–1^ (with a 2.00 sccm effluent flow rate and 5 cm^2^ electrode) is achieved and a sputter-deposited (S–Ru) catalyst is tested with a short test run time (<1 h) and a cost-effective amount of ^15^N_2_ (<100 mL). The S–Ru is found to show no detectable N_2_RR activity under the tested conditions. The demonstrated multiturn TOF-MS method and protocol address the need for NH_3_ analytical methods that are resistant to false positive results by circumventing the high background of ^14^NH_3_ and labile ^14^N-containing contaminant species and instead utilizing rapid, in-line ^15^NH_3_ quantification. This powerful method for N_2_RR catalyst testing in a scalable cell architecture provides the much needed traction for validating, investigating, and developing N_2_RR catalysts.

## Supporting Information Available

Discussion of the importance of maintaining a near-constant carrier gas N_2_/H_2_ ratio during catalyst testing, schematic diagram of N_2_RR test station, single-ion chromatograms showing ^15^NH_3_^+^ signal over time with a powder-absorbent-media gas purifier engaged, single ion chromatogram showing ^14^NH_3_^+^ signal resulting from background ^14^NH_3_ during the instrument stabilization and warm up period, X-ray photoelectron spectroscopy (XPS) characterization of sputter-deposited Ru (S–Ru) catalyst, details of XPS analysis of sputter-deposited Ru (S–Ru) catalyst, calibration curve showing ^14^NH_3_^+^ signal as a function of ^14^NH_3_ concentration, single-ion chromatogram showing ^15^NH_3_^+^ signal during S–Ru catalyst testing using multiple current holds of −28.6 mA·cm^–2^ allowing the composition of the carrier gas to vary, and single-ion chromatograms showing ^15^NH_3_^+^ signal and ^14^NH_3_ signal during S–Ru catalyst testing using multiple current holds of −28.6 mA·cm^–2^ (PDF)
