# Supplementary material for: Nitrogen Reduction Testing with Real-Time 15NH3 Yield Quantification Using Orbital Multiturn Time-of-Flight Mass Spectrometry
Source: ACS Energy Lett. 2024 Nov 8;9(12):5780–6. doi: 10.1021/acsenergylett.4c02961 (PMC11650762; doi:10.1021/acsenergylett.4c02961)
Supplement: Supplementary file 1 — nz4c02961_si_001.pdf [file nz4c02961_si_001.pdf]

Supporting Information

Nitrogen Reduction Testing with Real-Time $^{15}NH_3$ Yield Quantification using Orbital Multi-Turn Time-of-Flight Mass Spectrometry

Logan M. Wilder[1], Kabirat Balogun[1], W. Ellis Klein[1], Prithviraj Chumble[1], James L. Young[1]*

[1]National Renewable Energy Laboratory, Golden, CO 80401, USA

*Corresponding author email: james.young@nrel.gov

Table of Contents

	Page
Details of Experimental Procedure	S2
Discussion of the importance of maintaining a near-constant carrier gas N_2/H_2 ratio during catalyst testing	S3
Figure S1. Schematic diagram of N_2RR test station showing location of and connection between components and direction of gas flow	S4
Figure S2. Single-ion chromatograms showing $^{15}NH_3^+$ signal over time with a powder-absorbent-media gas purifier engaged	S5
Figure S3. Single ion chromatogram showing $^{14}NH_3^+$ signal resulting from background $^{14}NH_3$ during the instrument stabilization and warm up period	S6
Figure S4 X-ray photoelectron spectroscopy (XPS) characterization of sputter-deposited Ru (S-Ru) catalyst before and after Isotope Tracing Protocol testing showing mixed Ru^0 and RuO_x before electrolysis and mixed Ru^0 and RuO_x with a reduced RuO_x component after electrolysis	S7
Details of XPS Analysis of Sputter-Deposited Ru (S-Ru) Catalyst	S7
Figure S5. Calibration curve showing $^{14}NH_3^+$ signal as a function of $^{14}NH_3$ concentration	S8
Figure S6. Single-ion chromatogram showing $^{15}NH_3^+$ signal during S-Ru catalyst testing using multiple current holds of –28.6 $mA\cdot cm^{-2}$, allowing the composition of the carrier gas to vary.	S9
Figure S7. Single-ion chromatograms showing $^{15}NH_3^+$ signal and $^{14}NH_3$ signal during S-Ru catalyst testing using multiple current holds of –28.6 $mA\cdot cm^{-2}$, with the composition of the carrier gas maintained at a constant 50:50 mixture of $^{15}N_2$ and H_2 (t = 0–185 min) or a constant 50:50 mixture of $^{14}N_2$ and H_2 (t = 185–350 min)	S10

Details of Experimental Procedure

XPS characterization of S-Ru gas diffusion electrode (GDE)

The S-Ru GDE was characterized *ex-situ* using X-ray photoelectron spectroscopy (XPS) to determine the composition before and after testing. XPS was carried out in an ultrahigh vacuum system with analysis chamber equipped with Physical Electronics 5600 system with monochromatic Al kα X-ray source and hemispherical analyzer. XPS data was analyzed using CasaXPS software Version 2.3.23PRI.0.

Multi-turn time of flight mass spectrometry (TOF-MS) parameters

Unless otherwise specified, the TOF-MS (JEOL, model # JMS-MS3010HRGA) is operated with a turn number of 24, indicating an ion flight path length of about 24 m, and the electron impact (EI) ionization energy is 11.5 eV. The electron energy of 11.5 eV is significantly lower than the standard electron energy for routine EI mass spectrometry (70 eV). In EI, 70 eV electron energy results in significant fragmentation of chemical species, which is useful for generating fragmentation patterns to identify species, but 70 eV EI lowers the intensity of the parent ion and results in a high density of peaks in the low m/z range. Here, fragmentation at 70 eV is unnecessary as species of interest are identified by measuring their mass to within +/-0.001 amu rather than by their fragmentation pattern.

Discussion of the importance of maintaining a near-constant carrier gas N_2/H_2 ratio during catalyst testing

An important consideration in using electron impact (EI) ionization for NH_3 ionization during nitrogen reduction reaction (N_2RR) catalyst testing is that the matrix gas composition influences NH_3 ionization efficiency due to competitive ionization between the matrix and analyte. Typically, in N_2RR catalyst testing experiments, significant quantities of H_2 are generated during cell operation due to the hydrogen evolution reaction (HER) side reaction. Thus, in the case in which N_2 is supplied to the cathode-side flow field, at open circuit, the cell effluent is ~ 100% N_2. When the cell is in operation, varying amounts of H_2 in the cell effluent result from varying levels of current, assuming HER occurs as a side reaction. For example, the cell current density applied in this study, –28.6 mA·cm^{-2}, (143 mA total for the 5 cm^2 area used), produces 1.00 sccm H_2 assuming 100% faradaic efficiency towards HER. This dilutes the 1.00 sccm N_2 flow by 50%. The ionization efficiency of H_2 is lower than N_2, and therefore the amount of NH_3 ionized increases with increasing percentage of H_2 in the carrier gas. Calibration curves for NH_3 demonstrating the difference between a 100% N_2 background and a varying H_2 percentage in the background are shown in Figure S5. The NH_3 calibration curve measured under 100% N_2 background is linear. The curve with varying amounts of H_2, which results from operating the cell at different current densities, is non-linear.

In the current work, two experimental design parameters are used to control changes to the H_2 proportion in the carrier gas. First, the cell was operated galvanostatically, with the assumption that the catalyst tested, sputter-deposited Ru, shows high FE towards HER (>95%) and low FE towards N_2RR, an assumption which is confirmed as described in the Main Text. Second, in the Isotope Tracing Protocol (shown in Figure 3 of the Main Text), the cathode-side outlet gas is maintained as a 1:1 mixture of N_2 and H_2 prior to and after the galvanostatic current hold. Thus, a constant 1:1 H_2:N_2 is sustained throughout the test, eliminating the influence of a varying H_2:N_2 ratio on the measured NH_3 signal. In future catalyst tests using the system described here with catalysts showing higher FE towards N_2RR, the H_2 percentage of the carrier gas could be tracked in real time, and the appropriate N_2:H_2 calibration curve selected.

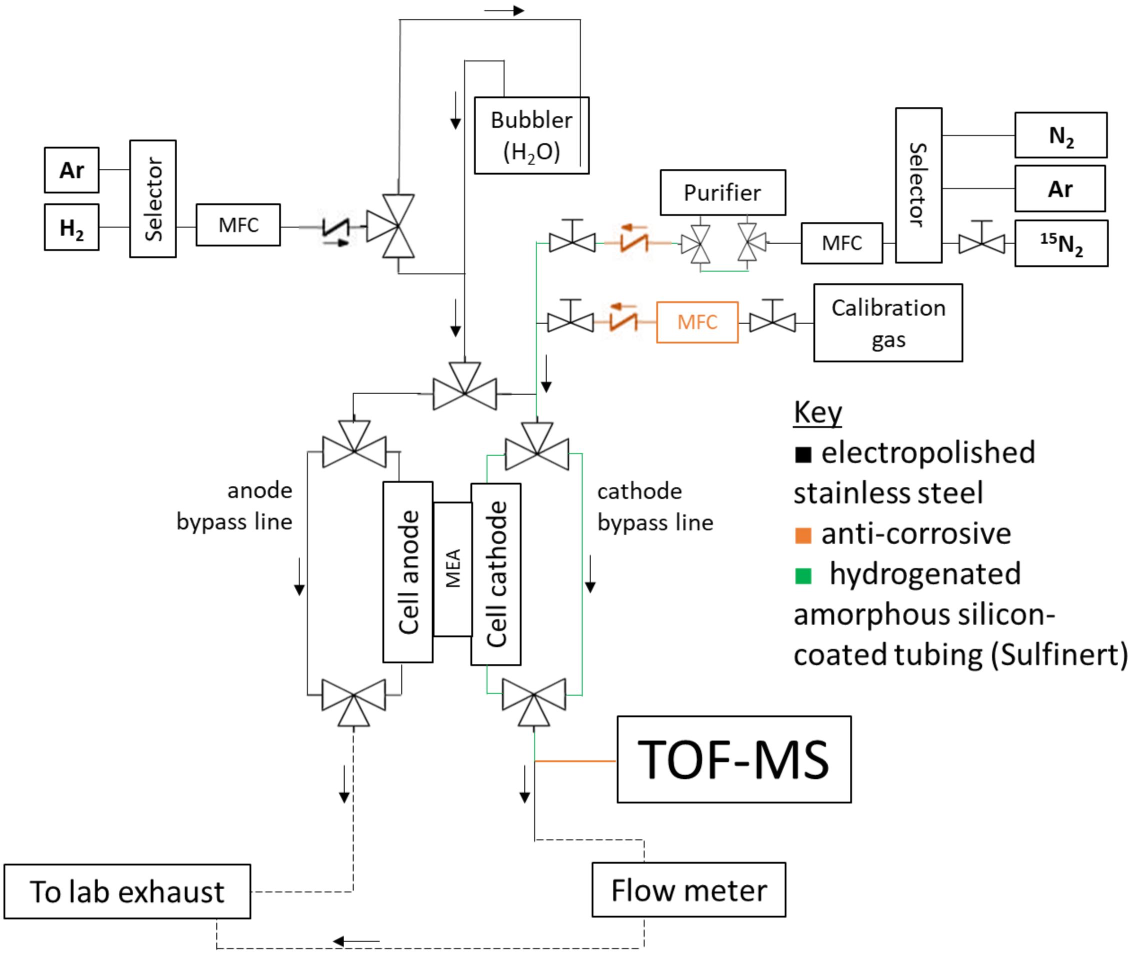

Figure S1. Schematic of N_2RR test station showing location of and connection between components and direction of gas flow.

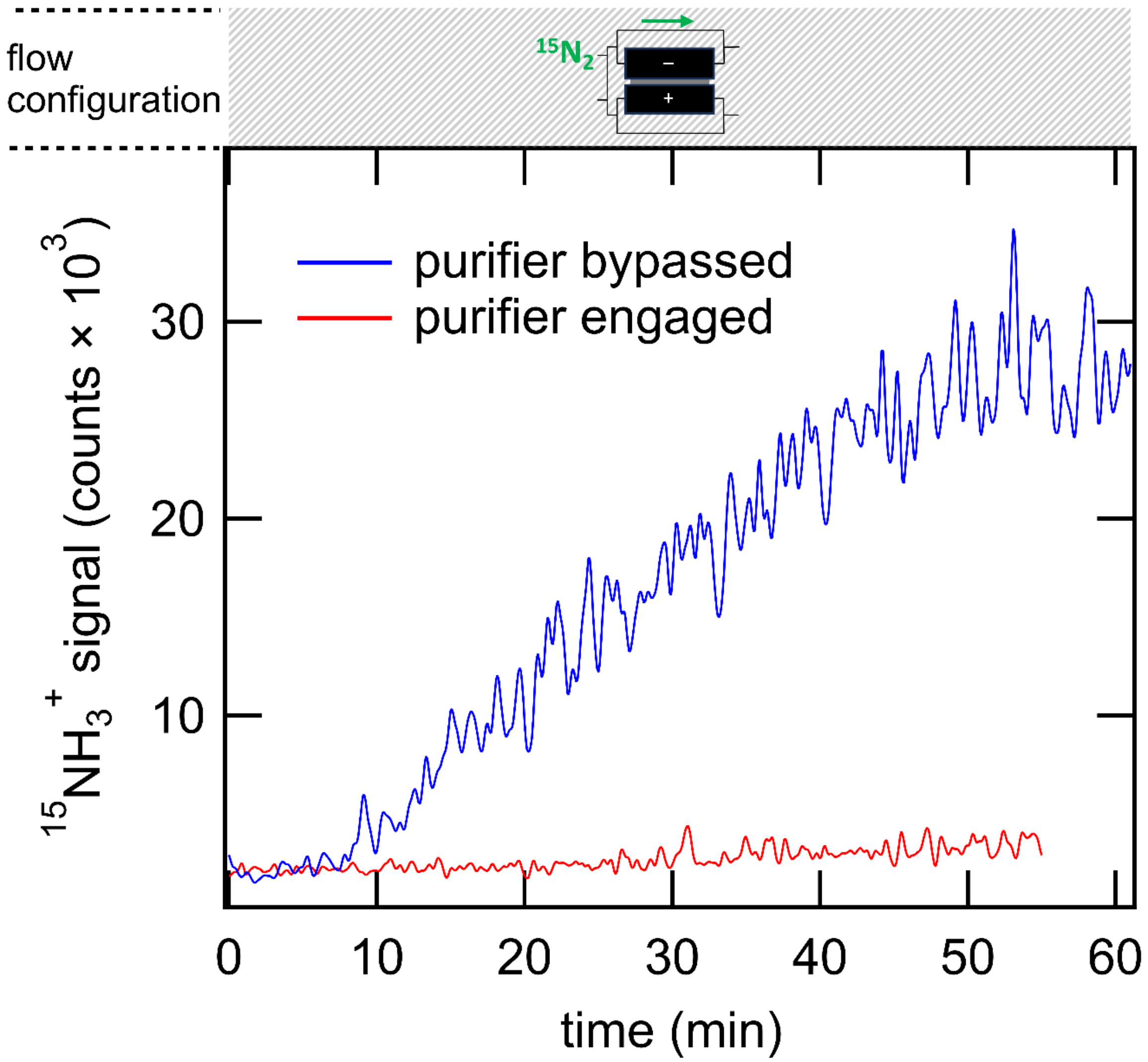

Figure S2. Single-ion chromatograms showing $^{15}NH_3^+$ signal over time with a powder-absorbent-media gas purifier engaged (UltraPure Mini PF, NuPure) (red trace) or bypassed (blue trace).

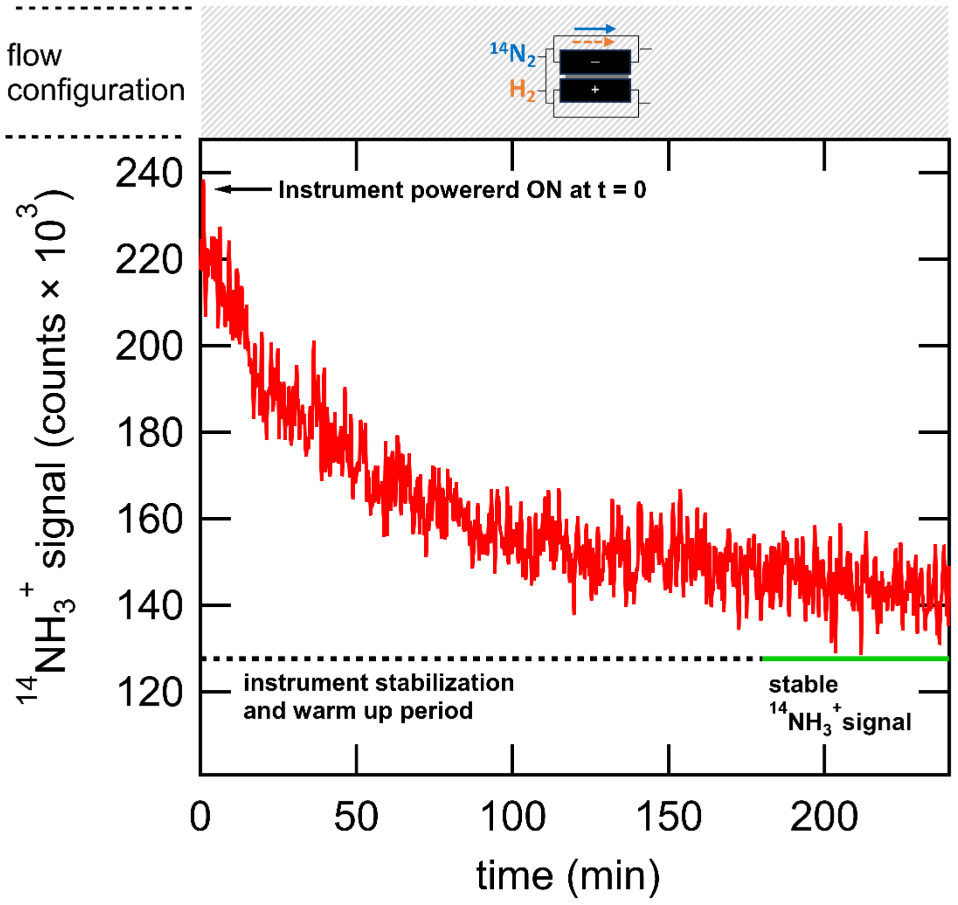

Figure S3. Single ion chromatogram showing $^{14}NH_3^+$ signal resulting from background $^{14}NH_3$ during the instrument stabilization and warm up period. A stable signal is defined as <2% average$_{30\ min}$ signal change over 1 h.

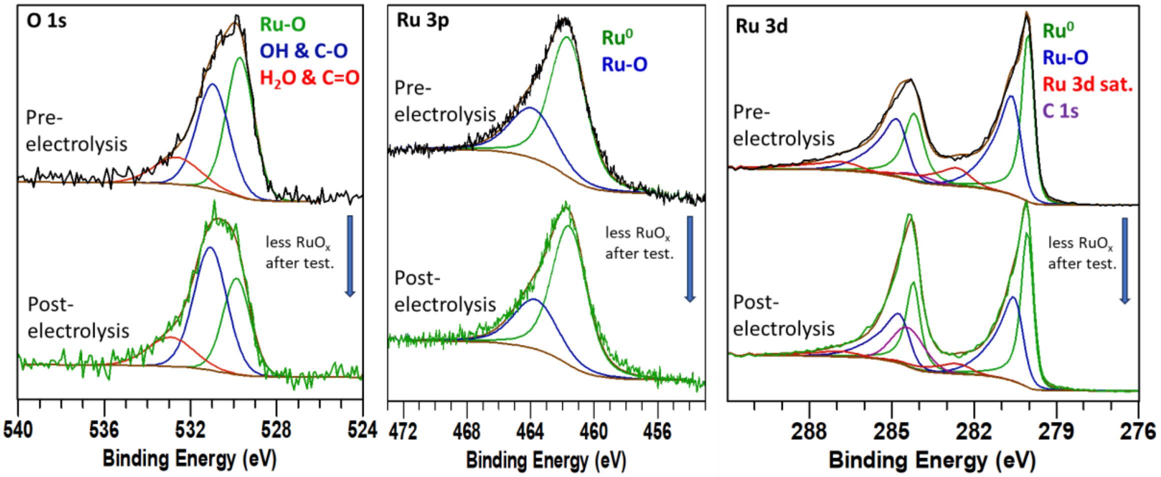

Figure S4. X-ray photoelectron spectroscopy (XPS) characterization of sputter-deposited Ru (S-Ru) catalyst before and after Isotope Tracing Protocol testing showing mixed Ru^0 and RuO_x before electrolysis and mixed Ru^0 and RuO_x with a comparatively reduced RuO_x component after electrolysis.

Details of XPS analysis of sputter-deposited Ru (S-Ru) catalyst

Analysis of O 1s, Ru 3p, and Ru 3d core level spectra show that the as-deposited Ru surface is composed of Ru^0 mixed with RuO_x, which is a common occurrence in DC magnetron sputtered Ru thin films exposed to ambient conditions.[1] The Ru $3d_{5/2}$ component with binding energy (B.E) at 280.01 eV, with a corresponding Ru $3d_{3/2}$ at 284.18 eV, are assigned to metallic Ru. This B.E in addition to the peak splitting difference of 4.17 eV closely agrees with those reported in literature.[1–3] The RuO_x component at 280.51 eV and 294. 68 eV corresponds to RuO_2. The C 1s present at 284.5 eV overlaps with the Ru $3d_{3/2}$ peak while the doublet at 282.46 eV and 286.63 eV are the Ru 3d satellite peak associated with RuO_2. The Ru $3p_{3/2}$ peaks are at 461.5 eV and 463.8 eV, respectively, and correspond to Ru metal and RuO_2. After catalyst testing, the oxide component is reduced as observed in the reduction of oxide component of the Ru 3d core level spectra.

The Finite Lorentzian line shape was applied to the Ru 3d and 3p peaks. For Ru $3d_{5/2}$ and Ru $3d_{3/2}$, LF (0.8, 1.25, 500, 180) and LF (1.01, 1.25, 500, 50) were applied respectively for metallic Ru while LF (0.25, 1,45, 280) was applied to the RuO_2 oxide components. The Gaussian–Lorentzian (GL (30)) peak shape was employed to fit the O 1s peak.

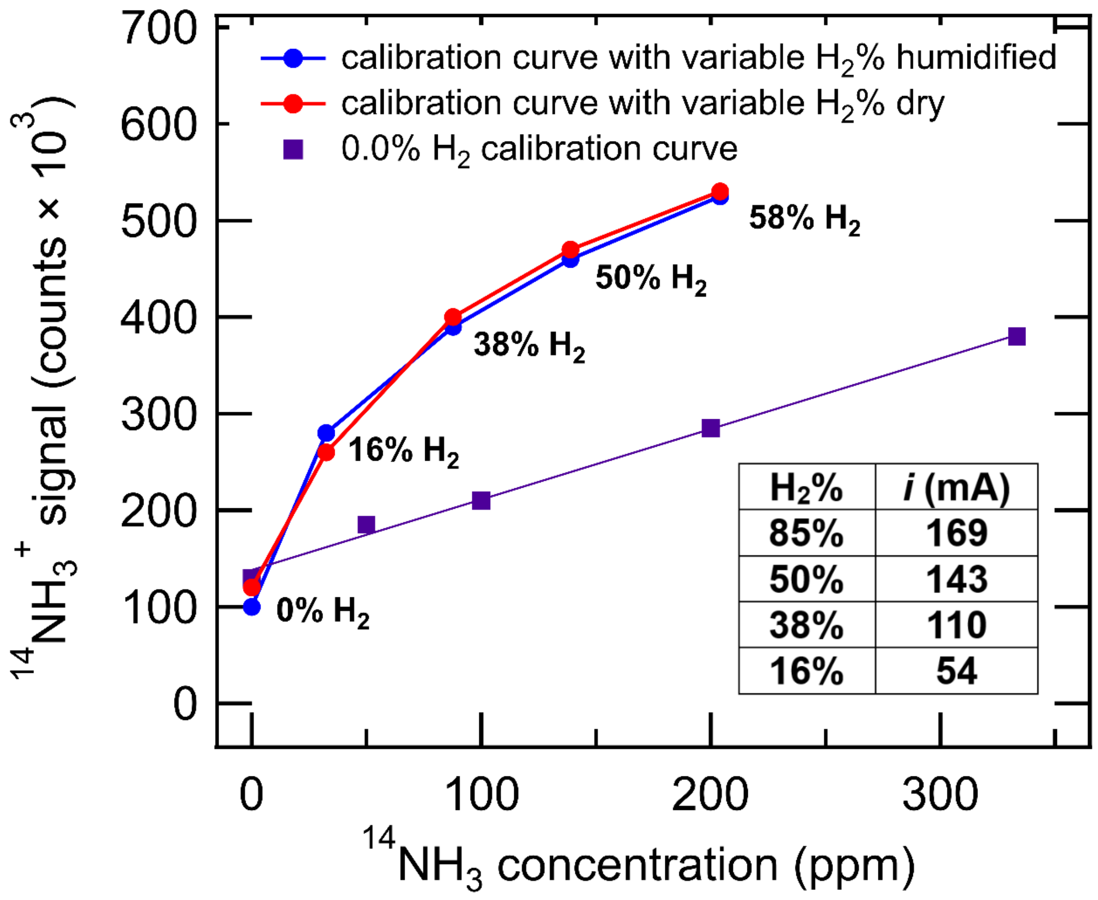

Figure S5. Calibration curve showing $^{14}NH_3^+$ signal as a function of $^{14}NH_3$ concentration. The purple curve represents 100% N_2 carrier gas. The red curve represents a calibration curve in which increasing NH_3 is accompanied by increasing H_2 as a percentage of the carrier gas (without humidification), and the blue curve shows the same with humidification.

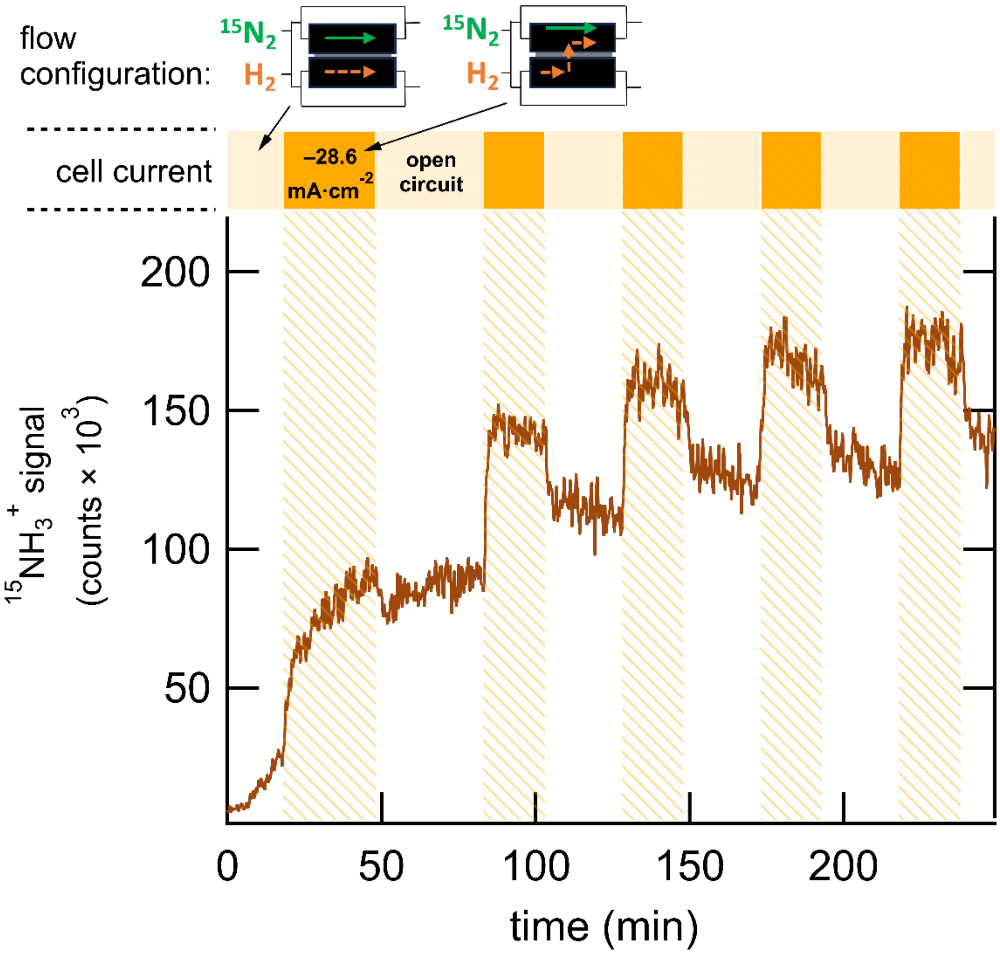

Figure S6. Single-ion chromatogram showing $^{15}NH_3^+$ signal during S-Ru catalyst testing using multiple current holds of –28.6 mA·cm^{-2}, allowing the composition of the carrier gas to vary. The composition of the carrier gas at open circuit is primarily $^{15}N_2$, and during the current holds, the composition of the carrier gas is a 50:50 mixture of $^{15}N_2$ and H_2 due to H_2 produced by the HER side reaction. The spikes in signal during the current holds are due to the variation in carrier gas composition and are not an indication of N_2RR activity.

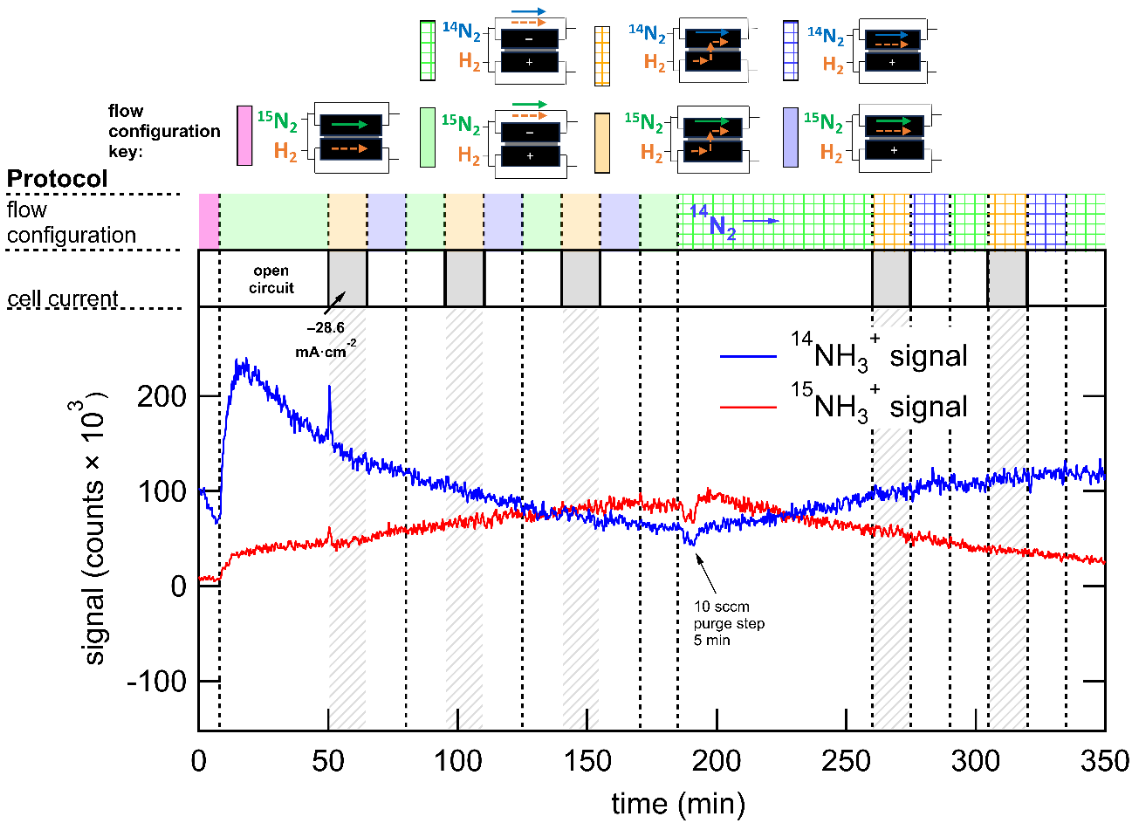

Figure S7. Single-ion chromatograms showing $^{15}NH_3^+$ signal (red) and $^{14}NH_3$ signal (blue) during S-Ru catalyst testing using multiple current holds of –28.6 mA·cm^{-2}, with the composition of the carrier gas maintained at a constant 50:50 mixture of $^{15}N_2$ and H_2 (t = 0–185 min) or a constant 50:50 mixture of $^{14}N_2$ and H_2 (t = 185–350 min). The total cathode flow rate during the run was 2.00 sccm except for t = 185–190 min when the flow rate was increased to 10.0 sccm to facilitate purging of the test station.

References

(1) Bianchi, C. L.; Ragaini, V.; Cattania, M. G. An XPS Study on Ruthenium Compounds and Catalysts. *Materials Chemistry and Physics* **1991**, *29* (1–4), 297–306. https://doi.org/10.1016/0254-0584(91)90025-P.

(2) Morgan, D. J. Resolving Ruthenium: XPS Studies of Common Ruthenium Materials. *Surface & Interface Analysis* **2015**, *47* (11), 1072–1079. https://doi.org/10.1002/sia.5852.

(3) Diulus, J. T.; Tobler, B.; Osterwalder, J.; Novotny, Z. Thermal Oxidation of Ru(0001) to RuO_2 (110) Studied with Ambient Pressure x-Ray Photoelectron Spectroscopy. *J. Phys. D: Appl. Phys.* **2021**, *54* (24), 244001. https://doi.org/10.1088/1361-6463/abedfd.
